# Gut Microbiome Variations in Herring Gulls (*Larus argentatus*) from Different Environments in the United Kingdom

**DOI:** 10.3390/ani16020300

**Published:** 2026-01-19

**Authors:** Wai Tung Kan, Samantha A. Siomko, Nicola J. Rooney, Paul Wigley

**Affiliations:** Bristol Veterinary School, University of Bristol, Bristol BS40 5DU, UK; vivienne.k@protonmail.com (W.T.K.); jf23062@bristol.ac.uk (S.A.S.); paul.wigley@bristol.ac.uk (P.W.)

**Keywords:** gut microbiome, wild birds, urbanisation, captivity, anthropogenic perturbations, 16S amplicon sequencing, Herring Gulls

## Abstract

Over the last century, anthropogenic activities have contributed to habitat degradation and fragmentation but have also affected the individual health of animals. In this study, we investigated the effect of environmental differences on the gut microbiome of Herring Gulls (*Larus argentatus*) by collecting fresh faecal samples from ten geographically different populations in the UK, including captive and wild birds, and comparing the resulting gut microbiome diversity and composition. A significantly higher alpha diversity was identified in captive gulls than in urban and suburban gulls for the 46 sequenced samples. When comparing gut microbiome composition, urban inhabitants exhibited a higher abundance of *Ligilactobacillus* and a lower abundance of *Streptococcus* than suburban gulls. Such differences could suggest a highly polluted environment for urban-dwelling gulls, while suburban populations could have a wider foraging range and a more diverse diet. In addition, samples from Bristol, West Kirby, Gloucester and Liverpool were all characterised by a significantly higher abundance of one or more of the other bacterial taxa. The high proportion of *Mycoplasma* could indicate avian mycoplasmosis in the Liverpool population. This study sheds light on the understudied subject of the wild avian gut microbiome and its possible application to wildlife health and disease management.

## Simple Summary

In recent years, scientists have been actively seeking non-invasive approaches to monitoring the health and welfare of wildlife. The Herring Gull is a highly adaptable species, which offers an excellent opportunity to study various potential environmental influences, including captivity, level of urbanisation, habitat types and geographical location. Here, we utilised faecal samples from different herring gull populations to analyse variation in their gut microbiome—the bacterial community residing in the guts. Populations sampled for this study include Bristol, Gloucester, Weston Super-Mare, Portishead, Hinkley Point, Steepholm Island, Hereford Wildlife Rescue Centre, Liverpool, Swansea and West Kirby. Significant differences were found between individual populations. Higher gut microbiome diversity (number of bacterial species) was identified in the captive population and in gulls from the city of Bristol. Moreover, several bacterial taxa were found to have significant differences in abundance when compared between areas and levels of urbanisation. Differences may be driven by factors such as higher pollution in urban areas, varied foraging ranges, and disease outbreaks, such as the possibility of Avian mycoplasmosis, an infectious respiratory disease, in the Liverpool population. These results suggest a need for further investigation into the effects of urbanisation on the health and welfare of wild animals. They demonstrate the applicability of gut microbial analysis to wildlife health monitoring at the population level and zoonotic disease control for the safety of both humans and animals.

## 1. Introduction

With the ongoing growth of human populations and the expansion of cities, natural environments are inevitably altered for agricultural, industrial and other purposes. Related consequences include the destruction and degradation of natural habitats and subsequent environmental pollution, climate change and rapid species extinction [1]. Also, numerous studies on the ecosystem and wildlife population have revealed negative effects of urbanisation [2]. It is, therefore, important to consider the possible impact of human activities on the internal health of wild animals, which is often overlooked and understudied. This is especially true for species adapted to live alongside humans and highly mobile species, like birds, as their foraging range often overlaps with human settlements. However, even species that tend to remain in their natural habitats suffer unavoidable changes like habitat fragmentation, landscape transformations and pollution.

The microbiome can be influenced by habitat disturbances [3,4], and analysis of the gut microbiome as a way to understand animal health has been gaining attention from researchers [5]. These bacterial communities inhabiting the host gastrointestinal (GI) environment, namely the gut microbiome, are found to aid in physiological functions like digestion, nutrient absorption and immunity, as well as cognitive abilities and behavioural processes [6]. Although current research efforts focus mostly on mammals and domesticated birds, it is found that both genetic factors [7,8] and external factors, like diet [9,10] and environment [11,12], contribute to wild avian gut microbiome diversity and composition. With over 10,000 wild avian species inhabiting diverse ecological niches, their gut microbiome health is vital for individual health and welfare, which is, in turn, essential for them to sustain their ecological roles. Moreover, given the migratory nature of most avian species, their wide geographical distribution and their potential as disease reservoirs [13], further understanding of their gut health could aid in disease control efforts.

In the few studies of how differences in environment contribute to wild birds’ gut microbiome changes, several environmental factors have been shown to have significant influences. The effects of factors including habitat [14,15], climate [16], diet [9,10], pollution [17,18] and captivity [19] have been assessed and revealed, mostly by comparative studies of different populations, while some incorporated experimental approaches. In one study of Swainson’s thrushes (*Catharus ustulatus*) and grey catbirds (*Dumetella carolinensis*), researchers compared the spring migrants with the fall migrants and found greater microbial compositional differences with seasonality than between species [20]. Hence, environmental factors appeared to have outweighed that of their host genetics for these two species. Other comparative studies on single species in different environmental conditions have also seen differences across various parameters, like alpha diversity, beta diversity and composition. For instance, the percentage of urban land coverage was shown to be associated with shifts in gut microbiome composition in American white ibises (*Eudocimus albus*) [13].

Most studies on avian species focus on passerines, while other species, like water birds, are largely unstudied [21]. The Herring Gull (*Larus argentatus*), a widely spread species across different habitats with abundant populations in the UK and Europe, is an ideal candidate for exploring the potential effect of environmental differences on the gut microbiome. This species is highly adapted to urbanised areas, living alongside humans, and along with some other sea birds, is on the UK Red List due to its rapid decline in number [22]. Comparative analysis between populations in locations with different urbanisation levels could provide insight into anthropogenic impact on their gut health [22]. Furthermore, urban gulls are considered a risk in the transmission of antimicrobial-resistant and zoonotic bacteria carried within the intestinal tract [23]. This study aims to compare and identify differences in the gut microbiome diversity and composition across Herring Gull populations living in locations with different degrees of urbanisation, in different geographic areas and also in captive settings.

## 2. Materials and Methods

### 2.1. Sample Collection

**Figure 1:**
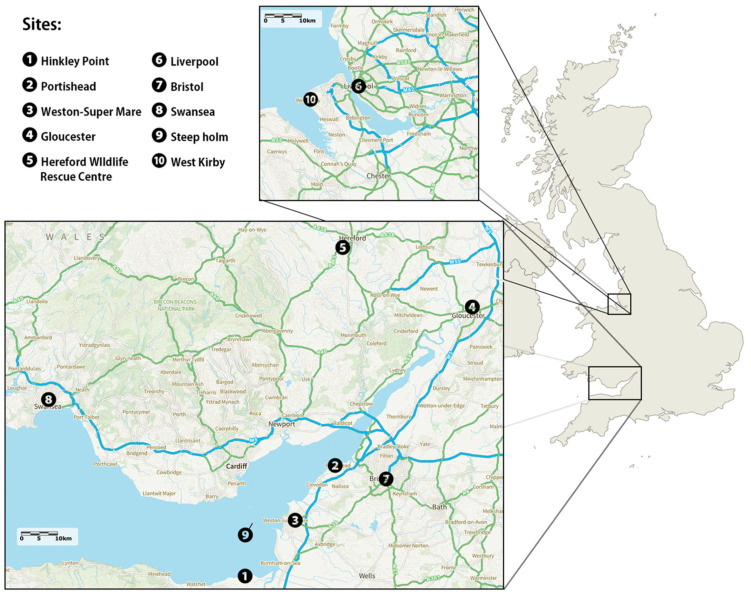
Sample collection locations (geographical locations of the 10 sample sites).

We sampled nine wild and one captive (Hereford Wildlife Rescue Centre HWRC) locations across Western UK (Figure 1). We obtained between 2 and 12 samples per site. At each site, between 1 and 10 h were spent observing gulls, resulting in a total of about 44 sampling hours. A total of 69 samples were collected between 25 June 2024 and 26 July 2024 and categorised by “Sites”, “Habitat” and “Sub-Habitat” according to the location of collection (Figure 1; Table 1). All wild samples were collected by direct observation of defecating Herring Gull individuals and immediate swabbing of faecal material using sterile collection swabs. Herring Gulls in the HWRC were separated into individual crates and were left alone for about 10–20 min prior to routine health checks. Faecal samples were collected from the crates using a sterile collection swab after returning the individuals to their enclosure. Juvenile individuals are indistinguishable between Herring Gulls and Lesser Black-backed Gulls (*Larus fuscus*), so sampled individuals seen alongside adult Herring Gulls were assumed to be juveniles of that species. Care was taken to avoid the white part of the droppings, which contains uric acid which can inhibit DNA extraction [24]. Faecal samples were placed in a sterile tube and stored in an insulated cool bag with ice blocks immediately after collection. All samples were placed in commercial freezers within 24 h of collection, then moved to −20 °C and finally −80 °C for long-term storage within a month until extraction.

### 2.2. DNA Extraction

DNA was extracted using the ZymoBIOMICS DNA Miniprep Kit (Zymo Research, Irvine, CA, USA) from 200 mg of thawed faecal material according to manufacturer’s protocol. The resulting DNA samples were eluted in 100 μL of ZymoBIOMICS^TM^ DNase/RNase Free Water. Extracted DNA was stored at 4 °C until sequencing. To quantify the DNA concentration, the Qubit dsDNA HS fluorometric kit (Thermo-Fisher Scientific, San Jose, CA, USA) was used. Considering the quality of the DNA samples, 46 out of 69 samples were chosen for PCR and sequencing.

### 2.3. Library Preparation for Nanopore Sequencing

The 16S Barcoding Kit 24 V14 (Oxford Nanopore Technologies, Oxford, UK) was used for 16S amplicon sequencing according to the manufacturer’s protocol. Samples were standardised to 10 ng DNA, and the entire 16S rRNA gene was amplified by PCR using primers 27F (5′-AGAGTTTGATCCTGGCTCAG-3′) and 1492R (5′-TACGGYTACCTTGTTACGACTT-3′) and LongAmp Taq 2x Master Mix (New England Biolabs, Ipswich, MA, USA).

The Agilent SureCycler 8800 was used for PCR and programmed as described in the barcoding kit. DNA concentration of the resulting amplified samples was quantified using the Qubit dsDNA HS fluorometric kit (Thermo-Fisher Scientific, San Jose, CA, USA), and all barcoded samples were pooled in varying volumes to contain a maximum of 75 ng DNA, according to individual concentration.

### 2.4. Nanopore Sequencing

The MinION flow cells by Oxford Nanopore Technology, Oxford, UK were primed and loaded for sequencing following the manufacturer’s protocol for the corresponding kit (16S Barcoding Kit 24 V14). Sequencing was performed for 74 h with the MinKNOW application (24.02.16) using the default settings with the function “Barcode Balancing” to account for uneven DNA concentrations across samples. A positive control mock microbial community was not included in this study; therefore, taxonomic assignments were interpreted in the context of known limitations of nanopore-based 16S sequencing and species-level analysis was avoided to reduce uncertainty. Negative sequencing control did not contain a significant number of reads. Basecalling was carried out on the GridION using Oxford Nanopore’s basecalling software (Dorado 7.3.11). Basecalled FASTQ files were subsequently analysed using the Epi2Me platform.

### 2.5. Taxonomy Assignment

EPI2ME software version 5.1.14 (Oxford Nanopore Technologies, Oxford, UK) was used to analyse the FASTA files with the wf-16s workflow and Minimap2 classifier. Reports were generated for alpha diversity metrics and OTU taxonomy abundance classified at the phylum to genus levels. Samples with the number of reads drastically lower (<1000) than the rest of the samples are not included in the data analysis, reducing the sample size to *n* = 40. Taxonomic richness and diversity metrics were calculated from unrarefied abundance tables generated by EPI2ME, and richness values were interpreted in a relative framework given sufficient sequencing depth across samples (Figure S1).

### 2.6. Data Analysis and Statistics

The alpha diversity metrics, including Pielou’s evenness, Berger-Parker index, Simpson’s Diversity Index, Inverse Simpson index, Shannon’s diversity index, total OTU count per sample, richness per sample and effective number of species, were considered. To analyse the microbial composition, abundance tables at the phylum, family and genus levels were devised. The taxa chosen for analysis were based on abundance and possible relevance (i.e., reported taxa related to external change from previous research papers).

Samples were categorised into “Sites”, “Habitat” and “Sub-Habitat” (Table 1). “Sites” refers to which sampling site the samples were from. Group definitions under the “Habitat” and “Sub-Habitat” categories are shown in Table 1. The groups “Rural”, “Town”, “Swansea” and “Steep-holm Island” were, however, excluded from any data analysis due to their extremely small group size (*n* = 1). However, all of the samples from these groups are still presented in bar charts. Apart from the sample of “Steepholm Island” and “Rural”, which is the same sample, other samples are still included in at least one of the ANOVA tests. For example, although “Swansea” is too small to be analysed as a “Site”, it still belonged to “Urban” when ANOVA tests were performed on “Habitats”. An additional category of “Age” is also applied to captive individuals to assess the age effect on the gut microbiome.

All statistical analyses were performed using R version 4.3.2. We tested for differences in alpha diversity metrics and taxa abundances between groups according to categories using ANOVA (Tables S1, S3, S5 and S7). When tested significant (*p* ≤ 0.05) or close to significant (*p* ≤ 0.10), post hoc Tukey’s Honest Significant Difference (HSD) test was performed with base R (Tables S2, S4, S6 and S8). The prevalence of *Mycoplasma* was calculated by considering the percentage of individual samples consisting of *Mycoplasma*. To visualise the microbial composition by phylum and family, stacked bar charts were created using Excel.

## 3. Results

Most of the results from ANOVA tests showed nonsignificant differences in alpha diversity metrics (Table 2 and Table S7). When comparing groups of “Sites”, there was a significant difference in the Inverse Simpson index. Further Tukey’s HSD tests suggested that “Bristol” had a significantly higher Inverse Simpson index than all the other sites (*p* < 0.05), with “West Kirby” being the most similar to “Bristol” (*p* = 0.0298) and “HWRC” being the most dissimilar (*p* = 0.0017) (Table S8).

When comparing “Habitats”, significant differences were identified in Shannon’s diversity index and the Effective number of species. Tukey’s HSD test reveals a significantly higher Shannon’s diversity index in “Captive” compared to both “Urban” (*p* = 0.0398) and “Suburban” (*p* = 0.0473). When considering the Effective number of species, the “Captive” population exhibited diversity when compared to the “Suburban” population (*p* = 0.028) only.

**Figure 2:**
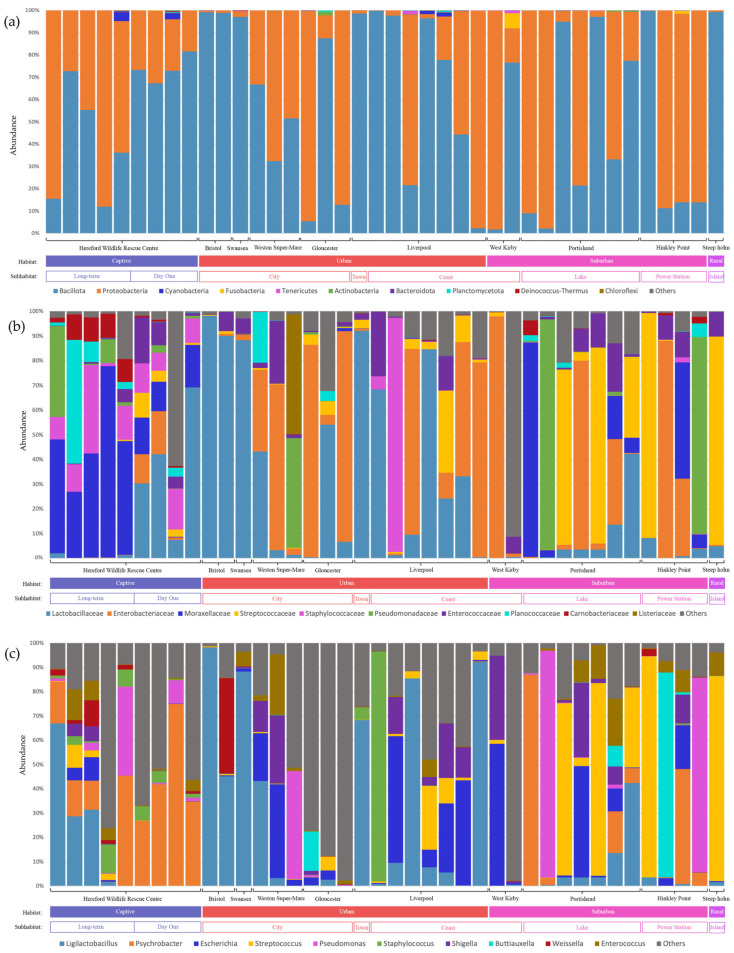
A stacked bar chart of the relative percentages of the 10 most numerous taxa of the Herring Gull gut microbial composition at the level of: (**a**) phylum; (**b**) family; (**c**) genus.

*Bacillota* and *Proteobacteria* showed the highest relative percentage of the total microbiome community at the phylum level (Figure 2a), but differences were much more pronounced at the family and genus level (Figure 2b,c).

Out of the seven phyla analysed (Table 3, Tables S1 and S2), significant differences were found between “Sites” in *Bacillota* (F = 2.468; *p* = 0.0399) and *Fusobacteriota* (F = 2.835; *p* = 0.0216). “West Kirby” displayed a significantly higher abundance of *Fusobacteriota* than did “Liverpool” (*p* = 0.0166), “Hereford Wildlife Rescue Centre” (*p* = 0.008), “Gloucester” (*p* = 0.0306), “Portishead” (*p* = 0.01) and “Weston Super-mare” (*p* = 0.0306). For *Bacillota*, however, there was no significant difference despite an observable trend for a higher abundance in the “Bristol” population (0.09 > *p* > 0.05).

*Staphylococcaceae* was the only family that differed significantly between “Habitat” (F = 4.533; *p* = 0.0175) with a higher abundance in “Suburban” than “Urban” population (*p* = 0.0226) and between “Sub-Habitat” (F = 2.592; *p* = 0.0446; Table 3, Tables S3 and S4). Although the “Lake” population showed a trend of higher abundance than that of “City” (*p* = 0.0501) and “Coast” (*p* = 0.0582), Tukey’s HSD test showed that no two sub-habitats differed significantly (Table S4). Since “Site” differences were close to significant (*p* = 0.0657), Tukey’s HSD test was carried out for the abundance differences in the *Lactobacillaceae* family. The “Gloucester” population had a higher abundance than “Liverpool” (*p* = 0.0228), “Portishead” (*p* = 0.0357) or “HWRC” (*p* = 0.0306) (Table S4).

When investigating the inter-group difference in abundance at the genus level, we found several significant differences across the “Sites”, “Habitats” and “Sub-Habitats” categories (Tables S5 and S6). Differences between “Sites” were identified in the genera *Bacillus* (F = 2.557; *p* = 0.0343), *Ligilactobacillus* (F = 2.736; *p* = 0.0254), *Klebsiella* (F = 5.777; *p* = 0.000266) and *Mycoplasma* (F = 2.49; *p* = 0.0178). Tukey’s HSD tests showed a significantly more abundant *Bacillus* bacteria community in the “Gloucester” site than in “HWRC” (*p* = 0.0113), “Hinkley Point” (*p* = 0.033) and “Liverpool” (*p* = 0.0244) (Table S6). The “Gloucester” population also showed a higher abundance of *Klebsiella* compared to all other “Sites” (0.0007 < *p* < 0.006), with a highly significant difference from “HWRC” (*p* = 0.00007). The “Bristol” site had a higher abundance of *Ligilactobacillus* than “Gloucester” (*p* = 0.0497), “Hinkley Point” or “Liverpool”. It is noteworthy that *Mycoplasma* was present in only 12 out of 40 samples (30%), with individuals from Liverpool making up most of those (7 individuals out of 8).

Among different “Habitats”, the abundance of *Ligilactobacillus* varied significantly (F = 3.466; *p* = 0.042), as did *Streptococcus* (F = 3.475; *p* = 0.0417) (Table S6). The “Urban” samples had a significantly higher abundance of *Ligilactobacillus* than the “Suburban” (*p* = 0.0387), whilst the “Suburban” population exhibited a nonsignificant trend towards higher *Streptococcus* than the “Urban” (*p* = 0.05001).

Genera abundances of *Acinetobacter* (F = 2.684; *p* = 0.039) and *Mycoplasma* (F = 2.557; *p* = 0.0469) also varied significantly between different “Sub-Habitats”(Table S6). The “Long-term” captive population had a more abundant *Acinetobacter* community than the “City” (*p* = 0.0424) and “Coast” populations (*p* = 0.0350), whilst Tukey’s HSD results found no significant difference in *Mycoplasma* between Sub-Habitats.

## 4. Discussion

This study aimed to identify the possible differences in gut microbiome diversity and composition between Herring Gull populations from various environments. Although the gut microbiome of all samples was dominated by *Bacillota* and *Proteobacteria*, we found differences in alpha diversity indexes and the microbial composition between sites and habitats. These findings align with most other studies around the impacts of external factors on avian gut microbiome, including habitat types, diet [9,10], pollution [25,26], and captivity [27]. Our results suggest the possible impact of captivity, urbanisation and geographical differences on avian gut microbiome.

### 4.1. The Effect of Captivity

When comparing the sequencing results of captive Herring Gulls with other free-roaming populations from different sites, we found a significantly higher Shannon’s diversity in the captive population than in urban and suburban populations. As one of the most used and effective diversity indexes, this finding suggests that Herring Gulls under captivity have a higher microbiome diversity. Another study also reported a similar result comparing a group of Common Kestrels (*Falco tinnunculus*) prior to and during captivity [27]. However, in contrast, a study of oriental white storks (*Ciconia boyciana*) [19] saw a less diverse gut microbiome in captive compared to wild birds [19]. Differences in diet, enclosure cleanliness, and size and other variables could explain the contrasting results. The association between diet and the wild avian gut microbiome is relatively well-established [9,21,28,29,30]. At our captive sampling location, the HWRC, gulls were fed a mix of dog food, chicken and fresh fish. This diet is likely to be higher in protein and dietary fibre than that of the Herring Gulls living near human settlements [31] who scavenge on human leftovers like chips, bread and other processed foods, which are low in MACs (microbiota-accessible carbohydrates), complex carbohydrates found in dietary fibre [32]. MACs are crucial carbon and energy sources for distal gut microbiota, and hence influential for the microbial community [32], which could explain the higher diversity of their gut microbiome in captivity. Similarly severe and long-lasting reductions in gut microbial diversity were observed in many studies on mammals with a low-MACs diet (processed food, high in fat and low in fibre) [32].

A higher abundance of *Acinetobacter* was also identified in the “Long-term” group, which are rescued gulls that were kept in enclosures for more than a year. There has been increasing attention on the genus *Acinetobacter* due to its pathogenic properties and exceptional ability to develop drug resistance, especially the species *Acinetobacter baumannii* [33]. However, according to a study performed on black-headed gulls and songbirds, *A. baumannii* did not show a preference for avian hosts [33]. Since the genus *Acinetobacter* is not uncommon in soil, water and humans, including healthy individuals [34], the higher frequency and proximity of interactions with humans in a captive setting might have caused the enriched population of this genus.

### 4.2. The Effect of Urbanisation

One of the main objectives of this study was to identify any associations between urbanisation level and gut microbial shifts. Although no differences were identified in alpha diversity, the “Urban” population had a significantly higher abundance of *Ligilactobacillus*. Being a genus of lactic acid bacteria, *Ligilactobacillus* spp. play important roles in food digestion, absorption, and strengthening host resistance to pathogens, especially the species *Ligilactobacillus salivarius* [35]. An increase in their number could be a mitigating strategy against the toxic effects of polluted environments. This has been suggested in another study of the impact of heavy metal exposure on tree sparrows (*Passer montanus*), with an increase in *Lactobacillus* being one of the significant findings [17]. *Lactobacillus* and *Ligilactobacillus* are taxonomically very close and were classified, along with 24 other genera, as one genus until 2020 [36].

On the other hand, the bacterial family *Staphylococcaceae* from the phylum *Bacillota*, which is generally characterised by its digestion-aiding ability, had a higher abundance in “Suburban” groups than both “Urban” and “Captive” populations. This family is ubiquitous in bird microbiota, with a few exceptions of infectious species like *Staphylococcus aureus* [37]. Our finding is corroborated by studies on house sparrows in which the urban individuals exhibited lower *Staphylococcaceae* abundance [10,38].

The abundance of the genus *Streptococcus* was marginally higher in the “Suburban” population than in the “Urban” population. This might indicate the more diverse diet of the Herring Gulls inhabiting “Suburban” sites, as in a previous large-scale study, omnivorous wild birds were found to have a higher gut content of *Streptococcus* than granivorous species [39]. Observationally, active feeding by humans is more frequent in the “Urban” sites than in the “Suburban” sites. Although further research is needed for Herring Gulls, a study on anthropogenic feeding on yellow-legged gulls has highlighted bread as the most commonly fed item, which is made from grains [40].

### 4.3. Site-Specific Characteristics

We saw that the gut microbiome of birds from some of the study sites stood out from the others. First, “Bristol” is the only group with significant alpha diversity differences compared to other sites, as reflected by the Inverse Simpson index. This indicates a higher gut microbial diversity with an emphasis on species evenness. Considered as a highly urbanised site, “Bristol” inhabitants might be exposed to a higher diversity of pathogenic and toxin-related bacteria, resulting in a higher alpha diversity. The heightened diversity is accompanied by a significantly higher abundance of the phylum *Bacillota* and the family *Ligilactobacillus*, which could be a mitigating strategy, as discussed above. Although *Bacillota* are commonly regarded as beneficial bacteria, the domination of this phylum could be a result of the high-fat content in the diet, as recognised in human and rodent studies [41]. The overall characteristics of this site could possibly suggest a highly polluted environment with high-fat food made available by humans, which coincides with our observation of this study location. However, it is noteworthy that similar situations could also be observed in other urban sites, and therefore, this might not be the only reason behind the significant results. Likewise, several detected genera, including *Bacillus* and *Clostridium*, are spore-forming taxa that are widely distributed in the environment and commonly encountered as laboratory or reagent contaminants. Detection of these genera may represent transient individuals instead of part of the established stable gut microbiome community. As such, quantification of *Bacillota* may represent artificially inflated counts of *Bacillus*. While we did not detect significant amounts of either taxon in our negative controls, we cannot definitively distinguish environmental introduction from true colonisation.

Secondly, “Gloucester” samples showed significantly higher bacterial content of the family *Lactobacillaceae* and the genera *Bacillus* and *Klebsiella*. High abundance of *Lactobacillaceae* in more urbanised avian populations has also been recorded in two studies on house sparrows [10,38]. The genus *Lactobacillus*, *Lactobacillaceae* includes many probiotic species crucial for intestinal health, regulating the immune system and protecting against infections [42]. While it is generally believed that an abundance of probiotic-implicated taxa indicates healthy individuals, a strikingly higher abundance of *Klebsiella* was also found in this population. Although *Klebsiella* spp. are commonly isolated in the environment and also in the animal gastrointestinal tract, species like *Klebsiella pneumoniae* can lead to respiratory infections in birds [43]. This species is also highly dominant, making up 95% of the total *Klebsiella* spp. isolated from turkeys in a study [44]. Furthermore, the high presence of *Klebsiella* spp. in this gull population indicates the possibility of them acting as mobile reservoirs for the spread of *Klebsiella pneumoniae*. This could be of public health concern, as they are known to cause infections by the multidrug-resistant strains [45].

Thirdly, in the case of the “West Kirby” population, a significantly higher proportion of *Fusobacteriota* was identified. This result is in line with research on the gut microbiome of 13 migratory shorebird species [15] and one study on urban Herring Gull [15,46]. *Fusobacteriota* have been associated with a carnivorous and omnivorous diet, and since West Kirby is on the coast, a small town with access to human but also natural rocky and sandy shorelines, this may reflect that the Herring Gulls in West Kirby are likely to scavenge yet still have a wider range for foraging [46].

A higher proportion of *Mycoplasma* was detected in the “Liverpool” population. Interestingly, in a previous large-scale study of over 1000 birds of 50 species, including 16 Herring Gulls [47], this bacterial genus showed a high occurrence (~80–90%) [45], which suggests *Mycoplasma* species are a part of the common gull microbiome. Here we saw a much lower prevalence (30%). Among over 100 species of *Mycoplasma*, some are more pathogenic than others, like *Mycoplasma gallisepticum* and *Mycoplasma synoviae*, which cause avian mycoplasmosis, and most of the others are opportunistic pathogens [47]. While *M. gallisepticum* and *M. synoviae* were not detected in the previous study, it is suggested that there might be different *Mycoplasma* species unique to *Laridae* birds [47]. Overall, the high abundance of *Mycoplasma* in Liverpool Herring Gulls could suggest a possible outbreak of mycoplasmosis, although this may be detection of *Mycoplasma* species that are part of the normal microbiome. Therefore, further research is needed to parse the difference between a disease outbreak and a high prevalence of *Mycoplasma* colonisation as part of normal microbiome variation.

### 4.4. Limitations

In our study, we used opportunistic faecal sample collection as our sampling method, which was possible because of the abundance of Herring Gulls and their proximity to human residences. However, this method does not result in a high sample size relative to collection effort. This is especially apparent in the Steepholm Island, where only two samples were collected despite over seven hours of observation by four researchers. In addition, our sampling period overlapped with one of the hottest periods of the year, which could have led to dehydration and inactivity in birds. Our resulting sample size is smaller than ideal when compared to other wild avian gut microbial research. Because richness estimates can be influenced by sequencing depth, comparisons are restricted to overall trends rather than absolute differences between samples. Although long-read nanopore 16S sequencing can improve resolution, species-level classification remains sensitive to sequencing errors, primer bias, and reference database accuracy. All analysis were performed at the genus classification level or higher to avoid these uncertainties at the species level. Conclusions are based on higher-level taxonomic trends and relative differences between samples. Furthermore, our study was limited to a non-invasive sampling method, which could lead to a higher chance of contamination and a different representation of the host gut microbiome compared to intestinal mucosal samples [48].

## 5. Conclusions

Our study provides valuable insights into the possible influence of habitat location, types and settings on avian gut microbiome diversity and composition using Herring Gulls as the research subject. While the dominance of the *Bacillota* and *Proteobacteria* phyla remained consistent with other avian gut microbiome studies, differences could be found in some of the alpha diversity indices and microbial compositions between habitat types and sampling sites. Some of these differences are candidates for further investigation.

Firstly, the impact of captivity on the gulls might seem paradoxical, with a seemingly positive impact as a higher Shannon’s index could imply; however, there is a possible health concern regarding a higher abundance of *Acinetobacter*. Secondly, the higher abundance of *Ligilactobacillus* in the urban gulls might indicate an environment with more pollutants, such as heavy metals, raising concern about the possible adverse effect of pollution on wild birds. In addition, Bristol, one of the highly urban sites, displayed a higher abundance of *Bacillota*, which could be explained by the high-fat processed food that is readily available. Thirdly, the suburban gull populations could have a more diverse diet and a wider range for foraging and scavenging than the urban gulls, as implied by the higher abundance of *Streptococcus* in suburban gulls. Finally, four of our sampling sites, Bristol, West Kirby, Gloucester and Liverpool, displayed characteristic features identifying a high prevalence of *Mycoplasma*, which may suggest a possible outbreak of mycoplasmosis in Liverpool gulls.

Further investigation should focus on testing for particular pathogenic species, diet analysis, foraging patterns and conducting experiments focusing on possible factors, function and impact of certain microbes on the host’s health. Increased sample sizes would also add power to the analysis. A better understanding of how the avian gut microbiome interacts with the habitat, and the host’s health and behaviour, could aid conservation efforts in different aspects. It could be applicable in managing environmental disturbances, including pollution, controlling disease and possible zoonoses, and aiding health care for captive breeding gulls, especially vulnerable species like the relict gulls (*Larus relictus*) [49].

## Figures and Tables

**Table 1:** Categorization of samples. All landscapes were visually assessed for coverage of permeable surfaces and vegetation to determine analysis Group.

Categories	Groups	Definition
Habitats (~5 km diameter from sampling point)	Captive	Any individuals living in an enclosure under human care.
	Urban	Any individuals sampled from a city where the surroundings consist mainly (>90%) of man-made buildings, shopping malls, impermeable paths and surfaces.
	Suburban	Any individuals sampled from smaller cities and towns where the surroundings consist of at least 50% of permeable surfaces, natural and/or coastal habitat.
	Rural	Any individuals sampled from natural habitat consisting <5% of impermeable surface.
Sub-Habitats	Day One	Any captive individual sampled the day after admission to the rescue centre.
	Long-term	Any captive individual sampled at least one year after admission to the rescue centre.
	Power Station	Any individuals sampled from a power station.
	Lake	Any individuals sampled by a lake.
	Coast	Any individuals sampled in a coastal area.
	Town	Any individuals sampled in a town, which includes residential area outside city centre.
	City	Any individuals sampled inside city centre, characterised by the presence of shopping malls and high-streets.
Sites	Hinkley Point (*n* = 4)	A nuclear power station in Somerset located by the coast. Considering the largely man-made landscape and unique properties of a power station, such as the presence of chemical tanks; classed as “Suburban; Power station”.
	Portishead (*n* = 7)	Despite it being a coastal site, all of the sampled individuals were juvenile seen staying around the Marine Lake; classed as “Suburban; Lake”.
	Weston Super-Mare (*n* = 3)	Samples are taken from area near a large supermarket with highstreets nearby; classed as “Urban; City”.
	Gloucester (*n* = 3)	Samples are taken near Gloucester Quays, a shopping mall; classed as “Urban; City”.
	Hereford Wildlife Rescue Centre (HWRC) (*n* = 9)	Sampled individuals are rescued in the city of Hereford and brought to the centre as injured or orphaned birds. Depending on their length of stay; classed as “Captive; Long-term/Day One”.
	Liverpool (*n* = 8)	The fifth largest city in the UK; classed as “Urban” and “Coast/Town” depending on sampling location.
	Bristol (*n* = 2)	Samples from within the in-land city centre area, consisting of shopping malls, busy traffics and streets of bars and restaurants; classed as “Urban; City”
	Swansea (*n* = 1)	One sample was taken from the site of Swansea city centre; classed as “Urban; City”
	Steepholm Island (*n* = 1)	An island six miles offshore from the Weston-Super Mare.
	West Kirby (*n* = 2)	A coastal town, despite the presence of a big marine lake; classed as “Suburban; coast” since all samples are adult gulls which have a much wider foraging range.

**Table 2:** Summary of ANOVA results for alpha diversity indexes. Significant *p*-values marked with “*” for *p* < 0.05, and “**” for *p* < 0.01.

Diversity	Sites		Habitats		Sub-Habitats		Age (HWRC)	
	F_7,30_ Value	*p* Value	F_2,36_ Value	*p* Value	F_7,30_ Value	*p* Value	F_2,6_ Value	*p* Value
Pielou’s evenness	1.497	0.206	1.971	0.154	1.091	0.384	0.875	0.464
Berger–Parker index	1.428	0.231	2.22	0.123	1.268	0.302	1.431	0.31
Simpson’s diversity index	1.65	0.16	2.55	0.0921	1.102	0.378	0.991	0.425
Inverse Simpson index	3.449	0.00794 **	1.588	0.218	0.617	0.688	1.292	0.341
Shannon’s diversity index	1.628	0.166	3.878	0.0298 *	1.83	0.135	0.705	0.531
Total OTU count per sample	1.35	0.262	0.587	0.561	2.107	0.0902	0.207	0.819
Species richness	1.249	0.308	3.242	0.0507	1.459	0.231	0.33	0.731
Effective number of species	1.136	0.368	4.073	0.0254 *	2.314	0.0667	0.613	0.573

**Table 3:** Summary of ANOVA results for comparing microbial composition at the phylum, family and genus levels. Significant *p*-values were marked with “*” for *p* < 0.05, and “***” for *p* < 0.001.

Taxonomical Groups	Sites		Habitats		Sub-Habitats		Age (HWRC)	
	F_7,30_ Value	*p* Value	F_2,36_ Value	*p* Value	F_7,30_ Value	*p* Value	F_2,6_ Value	*p* Value
**Phylum**								
*Bacillota*	2.468	0.0399 *	3.012	0.0617	0.795	0.561	0.367	0.707
*Proteobacteria*	0.82	0.578	1.488	0.239	1.387	0.255	2.81	0.138
*Bacteroidota*	0.495	0.831	2.076	0.14	1.333	0.276	0.9	0.455
*Fusobacteriota*	2.835	0.0216 *	1.694	0.198	0.946	0.465	0.847	0.474
*Cyanobacteria*	0.471	0.848	1.863	0.17	1.209	0.327	0.857	0.471
*Tenericutes*	0.902	0.518	0.906	0.413	1.207	0.328	0.348	0.719
*Planctomycetota*	1.032	0.43	1.225	0.306	0.37	0.865	0.341	0.724
**Family**								
*Enterobacteriaceae*	0.345	0.926	1.024	0.37	0.577	0.717	1.009	0.375
*Lactobacillaceae*	2.174	0.0657	0.484	0.62	1.215	0.325	1.265	0.294
*Enterococcaceae*	0.784	0.606	0.936	0.401	1.232	0.317	1.281	0.29
*Staphylococcaceae*	1.743	0.137	4.533	0.0175 *	2.592	0.0446 *	0.97	0.389
**Genus**								
*Lactococcus*	0.899	0.52	1.916	0.162	1.296	0.29	1.563	0.284
*Staphylococcus*	0.487	0.837	0.529	0.594	0.612	0.691	1.587	0.28
*Streptococcus*	1.125	0.374	3.475	0.0417 *	1.763	0.149	1.256	0.35
*Acinetobacter*	0.714	0.661	3.002	0.0623	2.684	0.039 *	1.031	0.412
*Salmonella*	0.833	0.569	0.942	0.399	1.165	0.348	1.511	0.294
*Bacillus*	2.557	0.0343 *	0.749	0.48	0.801	0.557	2.326	0.179
*Enterococcus*	1.083	0.398	0.686	0.51	1.459	0.231	1.086	0.396
*Lactobacillus*	1.244	0.311	0.715	0.496	1.849	0.131	0.591	0.583
*Ligilactobacillus*	2.736	0.0254 *	3.466	0.042 *	1.704	0.162	5.057	0.0516
*Clostridium*	0.627	0.729	0.748	0.481	0.858	0.519	1.256	0.35
*Campylobacter*	0.812	0.584	1.031	0.367	1.009	0.428	1.008	0.42
*Escherichia*	2.015	0.0861	0.656	0.525	1.718	0.159	0.556	0.601
*Klebsiella*	5.777	0.000266 ***	1.18	0.319	1.222	0.321	2.513	0.161
*Yersinia*	0.482	0.84	1.145	0.33	0.767	0.581	2.332	0.178
*Mycoplasma*	2.49	0.0178 *	3.112	0.0567	2.557	0.0469 *	0.556	0.601

## Data Availability

The sequences are uploaded on NCBI SRA (accession number: PRJNA1363092).

## Supplementary Materials

The following supporting information can be downloaded at: https://www.mdpi.com/article/10.3390/ani16020300/s1, Table S1: ANOVA test results—Phylum; Table S2: Tukey’s Honest Significant Difference (HSD) test results—Phylum; Table S3: ANOVA test results—Family; Table S4: Tukey’s Honest Significant Difference (HSD) test results—Family; Table S5: ANOVA test results—Genus; Table S6: Tukey’s Honest Significant Difference (HSD) test results—Genus; Table S7: ANOVA test results—Alpha Diversity; Table S8: Tukey’s Honest Significant Difference (HSD) test results—Alpha Diversity; Figure S1: Species Richness Curves.
