# Supplementary material for: Gut Microbiome Variations in Herring Gulls (Larus argentatus) from Different Environments in the United Kingdom
Source: Animals (Basel). 2026 Jan 19;16(2):300. doi: 10.3390/ani16020300 (PMC12837678; doi:10.3390/ani16020300)

Supplemental Material for:

Gut microbiome variations in Herring Gulls (Larus argentatus) from different environments in the United Kingdom

Wai Tung Kan [1]

Samantha A Siomko [1]

Nicola J Rooney [1*]

Paul Wigley [1*]

[1] Bristol Veterinary School, University of Bristol, Bristol, United Kingdom

*Corresponding author's address:

nicola.rooney@bristol.ac.uk (N. R.)

paul.wigley@bristol.ac.uk (P. W.)

Supplemental Tables

Table S1. ANOVA test results - Phylum

ANOVA results for comparing microbial composition on phylum level. Significant p-values were marked with "*" for p<0.05, "**" for p<0.01 and "***" for p<0.001

Phylum		D.f.	Sum Sq	Mean sq	F value	*P*
Bacillota	Habitat	2	2.263e+10	1.131e+10	3.012	0.0617
	Residuals	36	1.352e+11	3.756e+09		
	SubHabitat	5	1.432e+10	2.865e+09	0.795	0.561
	Residuals	32	1.153e+11	3.604e+09		
	Site	7	5.330e+10	7.615e+09	2.468	0.0399*
	Residuals	30	9.258e+10	3.086e+09		
	Age (HWRC)	2	2.021e+09	1.011e+09	0.367	0.707
	Residuals	6	1.653e+10	2.755e+09		
Proteobacteria	Habitat	2	1.233e+10	6.163e+09	1.488	0.239
	Residuals	36	1.492e+11	4.143e+09		
	SubHabitat	5	2.809e+10	5.618e+09	1.387	0.255
	Residuals	32	1.296e+11	4.050e+09		
	Site	7	2.537e+10	3.625e+09	0.82	0.578
	Residuals	30	1.326e+11	4.419e+09		
	Age (HWRC)	2	1.707e+10	8.534e+09	2.81	0.138
	Residuals	6	1.822e+10	3.037e+09		
Bacteroidota	Habitat	2	239420	119710	2.076	0.14
	Residuals	36	2075708	57659		
	SubHabitat	5	398686	79737	1.333	0.276
	Residuals	32	1914757	59836		
	Site	7	239636	34234	0.495	0.831
	Residuals	30	2074536	69151		
	Age (HWRC)	2	477744	238872	0.9	0.455
	Residuals	6	1592336	265389		
Fusobacteriota	Habitat	2	718303	359152	1.694	0.198
	Residuals	36	7630588	211961		
	SubHabitat	5	1075410	215082	0.946	0.465
	Residuals	32	7273475	227296		
	Site	7	3317514	473931	2.835	0.0216*
	Residuals	30	5015973	167199		
	Age (HWRC)	2	1780	889.9	0.847	0.474
	Residuals	6	6306	1050.9		
Cyanobacteria	Habitat	2	5155189	2577595	1.863	0.17
	Residuals	36	49812712	1383686		
	SubHabitat	5	8726581	1745316	1.209	0.327
	Residuals	32	46196852	1443652		
	Site	7	5432063	776009	0.471	0.848
	Residuals	30	49475657	1649189		
	Age (HWRC)	2	10602474	5301237	0.857	0.471
	Residuals	6	37109054	6184842		

Tenericutes	Habitat	2	252436	126218	0.906	0.413
	Residuals	36	5013291	139258		
	SubHabitat	5	829043	165809	1.207	0.328
	Residuals	32	4396791	137400		
	Site	7	912081	130297	0.902	0.518
	Residuals	30	4333773	144459		
	Age (HWRC)	2	10.14	5.069	0.348	0.719
	Residuals	6	87.42	14.569		
Planctomycetota	Habitat	2	13168	6584	1.225	0.306
	Residuals	36	193419	5373		
	SubHabitat	5	11265	2253	0.37	0.865
	Residuals	32	194769	6087		
	Site	7	39950	5707	1.032	0.43
	Residuals	30	165877	5529		
	Age (HWRC)	2	4455	2227	0.341	0.724
	Residuals	6	39160	6527		

Table S2. Tukey's Honest Significant Difference (HSD) test results - Phylum

Bacillota

	diff	lwr	upr	*p adj*
SubUrban - Captive	-43758.55	-108720.330	21203.24	0.2397030
Urban - Captive	10167.10	-51589.084	71923.29	0.9148208
Urban - SubUrban	53925.65	-1269.892	109121.20	0.0566549

Table S3. ANOVA test results - Family

ANOVA results for comparing microbial composition on family level. Significant p-values were marked with “*” for p<0.05, “**” for p<0.01 and “***” for p<0.001

Family		D.f.	Sum Sq	Mean sq	F value	*P*
Enterobacteriaceae	Habitat	2	6.31e+09	3.155e+09	1.024	0.37
	Residuals	36	1.11e+11	3.082e+09		
	SubHabitat	5	9.634e+09	1.927e+09	0.577	0.717
	Residuals	32	1.069e+11	3.339e+09		
	Site	7	8.383e+09	1.198e+09	0.345	0.926
	Residuals	30	1.042e+11	3.473e+09		
	Age (HWRC)	2	6.223e+09	3.111e+09	1.009	0.375
	Residuals	6	1.111e+11	3.085e+09		
Lactobacillaceae	Habitat	2	2.975e+09	1.487e+09	0.484	0.62
	Residuals	36	1.105e+11	3.070e+09		
	SubHabitat	5	1.797e+10	3.593e+09	1.215	0.325
	Residuals	32	9.461e+10	2.956e+09		
	Site	7	3.800e+10	5.429e+09	2.174	0.0657
	Residuals	30	7.491e+10	2.497e+09		
	Age (HWRC)	2	7.453e+09	3.727e+09	1.265	0.294
	Residuals	6	1.060e+11	2.946e+09		

Enterococcaceae	Habitat	2	1.969e+08	98438704	0.936	0.401
	Residuals	36	3.785e+09	105129045		
	SubHabitat	5	6.386e+08	127728350	1.232	0.317
	Residuals	32	3.316e+09	103638771		
	Site	7	6.123e+08	87473386	0.784	0.606
	Residuals	30	3.347e+09	111556502		
	Age (HWRC)	2	2.645e+08	132225930	1.281	0.29
	Residuals	6	3.717e+09	103251977		
Staphylococcaceae	Habitat	2	7.440e+09	3.720e+09	4.533	0.0175*
	Residuals	36	2.954e+10	8.206e+08		
	SubHabitat	5	1.063e+10	2.126e+09	2.592	0.0446*
	Residuals	32	2.625e+10	8.204e+08		
	Site	7	1.066e+10	1.523e+09	1.743	0.137
	Residuals	30	2.622e+10	8.740e+08		
	Age (HWRC)	2	1.891e+09	945737912	0.97	0.389
	Residuals	6	3.509e+10	974752078		

Table S4. Tukey's Honest Significant Difference (HSD) test results - Family

Staphylococcaceae ~ Habitat

	diff	lwr	upr	*p adj*
SubUrban-Captive	29127.4444	-1235.403	59490.29	0.062281
Urban-Captive	-263.0261	-29127.593	28601.54	0.9997265
Urban-SubUrban	-29390.471	-55188.621	-3592.32	0.022558*

Staphylococcaceae ~ SubHabitat

	diff	lwr	upr	*p adj*
Coast-City	952.77778	-39947.357	41852.913	0.9999997
DayOne-City	-53.63889	-52191.285	52084.007	1
Lake-City	43713.6825	-10.40074	87437.766	0.0500829
LongTerm-City	468.91111	-47924.781	48862.603	1
PowerStation-City	16689.8611	-35447.785	68827.507	0.9241625
DayOne-Coast	-1006.4167	-53144.063	51131.23	0.9999999
Lake-Coast	42760.9048	-963.17852	86484.988	0.0582138
LongTerm-Coast	-483.86667	-48877.559	47909.825	1
PowerStation-Coast	15737.0833	-36400.563	67874.73	0.9399564
Lake-DayOne	43767.3214	-10613.821	98148.463	0.1737033
LongTerm-DayOne	522.55	-57679.362	58724.462	1
PowerStation-DayOne	16743.5	-44606.702	78093.702	0.9603102
LongTerm-Lake	-43244.771	-94047.565	7558.022	0.1321489
PowerStation-Lake	-27023.821	-81404.963	27357.321	0.6634488
PowerStation-LongTerm	16220.95	-41980.962	74422.862	0.9566791

Staphylococcaceae ~ Site

	diff	lwr	upr	*p adj*
Gloucester-Bristol	113243.667	-35177.67	261665.002	0.2411118
Hereford Wildlife Rescue Centre-Bristol	-2156.222	-129256.66	124944.216	1
Hinkley Point-Bristol	17263.75	-123541.09	158068.592	0.9999055
Liverpool-Bristol	-8052.375	-136589.02	120484.272	0.999999
Portishead-Bristol	-3957.286	-134317.24	126402.667	1
West Kirby-Bristol	12119.5	-150467.93	174706.927	0.9999968
Weston Super-Mare-Bristol	20051.333	-128370	168472.669	0.9998192
Hereford Wildlife Rescue Centre-Gloucester	-115399.89	-223791.51	-7008.271	0.0305563*
Hinkley Point-Gloucester	-95979.917	-220158.12	28198.282	0.2277951
Liverpool-Gloucester	-121296.04	-231368.25	-11223.833	0.022787*
Portishead-Gloucester	-117200.95	-229396.94	-5004.969	0.0356781*
West Kirby-Gloucester	-101124.17	-249545.5	47297.169	0.3705226
Weston Super-Mare-Gloucester	-93192.333	-225944.41	39559.745	0.3345299
Hinkley Point-Hereford Wildlife Rescue Centre	19419.972	-78282.91	117122.856	0.9977788
Liverpool-Hereford Wildlife Rescue Centre	-5896.153	-84899.44	73107.136	0.9999968
Portishead-Hereford Wildlife Rescue Centre	-1801.063	-83737.43	80135.298	1
West Kirby-Hereford Wildlife Rescue Centre	14275.722	-112824.72	141376.161	0.9999477
Weston Super-Mare-Hereford Wildlife Rescue Centre	22207.556	-86184.06	130599.174	0.9973141
Liverpool-Hinkley Point	-25316.125	-124880.18	74247.934	0.990034
Portishead-Hinkley Point	-21221.036	-123128.1	80686.027	0.9970277
West Kirby-Hinkley Point	-5144.25	-145949.09	135660.592	1
Weston Super-Mare-Hinkley Point	2787.583	-121390.62	126965.782	1
Portishead-Liverpool	4095.089	-80051.9	88242.077	0.9999998
West Kirby-Liverpool	20171.875	-108364.77	148708.522	0.9995135
Weston Super-Mare-Liverpool	28103.708	-81968.5	138175.917	0.9897898
West Kirby-Portishead	16076.786	-114283.17	146436.739	0.9999018
Weston Super-Mare-Portishead	24008.619	-88187.36	136204.603	0.9964808
Weston Super-Mare-West Kirby	7931.833	-140489.5	156353.169	0.9999997

Table S5. ANOVA test results - Genus

ANOVA results for comparing microbial composition on genus levels. Significant p-values were marked with "*" for p<0.05, "**" for p<0.01 and "***" for p<0.001

Genus		D.f.	Sum Sq	Mean sq	F value	*P*
Lactococcus	Habitat	2	4475073	2237536	1.916	0.162
	Residuals	36	42046207	1167950		
	SubHabitat	5	7797332	1559466	1.296	0.29
	Residuals	32	38491809	1202869		
	Site	7	8024696	1146385	0.899	0.52
	Residuals	30	38247553	1274918		
	Age (HWRC)	2	3326360	1663180	1.563	0.284
	Residuals	6	6382553	1063759		
Staphylococcus	Habitat	2	9.520e+08	476004047	0.529	0.594
	Residuals	36	3.242e+10	900505388		
	SubHabitat	5	2.910e+09	582090905	0.612	0.691
	Residuals	32	3.042e+10	950573903		
	Site	7	3.398e+09	485474863	0.487	0.837
	Residuals	30	2.993e+10	997670115		
	Age (HWRC)	2	68857405	34428702	1.587	0.28
	Residuals	6	130156913	21692819		
Streptococcus	Habitat	2	3.395e+09	1.697e+09	3.475	0.0417*
	Residuals	36	1.758e+10	4.884e+08		
	SubHabitat	5	4.530e+09	9.06e+08	1.763	0.149
	Residuals	32	1.645e+10	5.14e+08		
	Site	7	4.344e+09	620510993	1.125	0.374
	Residuals	30	1.655e+10	551641703		
	Age (HWRC)	2	91997118	45998559	1.256	0.35
	Residuals	6	219651743	36608624		
Pseudomonas	Habitat	2	1.234e+09	617229736	0.896	0.417
	Residuals	36	2.481e+10	689032953		
	SubHabitat	5	2.849e+09	569883725	0.789	0.565
	Residuals	32	2.310e+10	721837445		
	Site	7	2.642e+09	377480377	0.486	0.837
	Residuals	30	2.331e+10	776859870		
	Age (HWRC)	2	1.163e+09	581716330	0.79	0.496
	Residuals	6	4.418e+09	736380417		
Bacteroides	Habitat	2	2.82	1.4103	1.542	0.228
	Residuals	36	32.92	0.9145		
	SubHabitat	5	7.822	1.5643	1.795	0.142
	Residuals	32	27.889	0.8715		
	Site	7	3.21	0.4586	0.423	0.88
	Residuals	30	32.50	1.0833		
	Age (HWRC)	2	5	2.5	0.556	0.601
	Residuals	6	27	4.5		
Acinetobacter	Habitat	2	80838628	40419314	3.002	0.0623
	Residuals	36	484783124	13466198		

	SubHabitat	5	166918521	33383704	2.684	0.039*
	Residuals	32	397966095	12436440		
	Site	7	80669125	11524161	0.714	0.661
	Residuals	30	484205017	16140167		
	Age (HWRC)	2	123443842	61721921	1.031	0.412
	Residuals	6	359266756	59877793		
Salmonella	Habitat	2	486505	243252	0.942	0.399
	Residuals	36	9297457	258263		
	SubHabitat	5	1505493	301099	1.165	0.348
	Residuals	32	8268622	258394		
	Site	7	1590048	227150	0.833	0.569
	Residuals	30	8181955	272732		
	Age (HWRC)	2	3268	1634	1.511	0.294
	Residuals	6	6488	1081		
Microvirga	Habitat	2	6355	3178	1.244	0.3
	Residuals	36	91948	2554		
	SubHabitat	5	12690	2538	0.952	0.461
	Residuals	32	85304	2666		
	Site	7	13513	1930	0.686	0.683
	Residuals	30	84480	2816		
	Age (HWRC)	2	5884	2942	0.33	0.731
	Residuals	6	53468	8911		
Bacillus	Habitat	2	27514	13757	0.749	0.48
	Residuals	36	660820	18356		
	SubHabitat	5	76420	15284	0.801	0.557
	Residuals	32	610682	19084		
	Site	7	256285	36612	2.557	0.0343*
	Residuals	30	429619	14321		
	Age (HWRC)	2	478.1	239.1	2.326	0.179
	Residuals	6	616.7	102.8		
Enterococcus	Habitat	2	7.493e+07	37466889	0.686	0.51
	Residuals	36	1.967e+09	54647723		
	SubHabitat	5	3.754e+08	75079178	1.459	0.231
	Residuals	32	1.647e+09	51475233		
	Site	7	4.023e+08	57472427	1.083	0.398
	Residuals	30	1.592e+09	53055110		
	Age (HWRC)	2	173566452	86783226	1.086	0.396
	Residuals	6	479679851	79946642		
Lactobacillus	Habitat	2	2861	1430	0.715	0.496
	Residuals	36	72049	2001		
	SubHabitat	5	16768	3354	1.849	0.131
	Residuals	32	58028	1813		
	Site	7	16820	2403	1.244	0.311
	Residuals	30	57953	1932		
	Age (HWRC)	2	268.9	134.4	0.591	0.583

	Residuals	6	1366.0	227.7		
Ligilactobacillus	Habitat	2	1.882e+10	9.412e+09	3.466	0.042*
	Residuals	36	9.776e+10	2.716e+09		
	SubHabitat	5	1.639e+10	3.278e+09	1.704	0.162
	Residuals	32	6.156e+10	1.924e+09		
	Site	7	3.813e+10	5.447e+09	2.736	0.0254*
	Residuals	30	5.972e+10	1.991e+09		
	Age (HWRC)	2	4.805e+09	2.403e+09	5.057	0.0516
	Residuals	6	2.851e+09	4.751e+08		
Clostridium	Habitat	2	4006744	2003372	0.748	0.481
	Residuals	36	96443779	2678994		
	SubHabitat	5	11851237	2370247	0.858	0.519
	Residuals	32	88353693	2761053		
	Site	7	12815386	1830769	0.627	0.729
	Residuals	30	87545714	2918190		
	Age (HWRC)	2	1662914	831457	1.008	0.42
	Residuals	6	4951518	825253		
Campylobacter	Habitat	2	13191862	6595931	1.031	0.367
	Residuals	36	230232441	6395346		
	SubHabitat	5	33140008	6628002	1.009	0.428
	Residuals	32	210120063	6566252		
	Site	7	38741546	5534507	0.812	0.584
	Residuals	30	204377886	6812596		
	Age (HWRC)	2	278.9	139.44	1.553	0.286
	Residuals	6	538.7	89.78		
Helicobacter	Habitat	2	10776542	5388271	0.376	0.689
	Residuals	36	515343554	14315099		
	SubHabitat	5	6.44e+07	12879984	0.894	0.497
	Residuals	32	4.61e+08	14406012		
	Site	7	55947839	7992548	0.511	0.819
	Residuals	30	469440997	15648033		
	Age (HWRC)	2	1.25	0.625	0.556	0.601
	Residuals	6	6.75	1.125		
Escherichia	Habitat	2	1.324e+09	6.619e+08	0.656	0.525
	Residuals	36	3.633e+10	1.009e+09		
	SubHabitat	5	7.935e+09	1.587e+09	1.718	0.159
	Residuals	32	2.956e+10	9.238e+08		
	Site	7	1.200e+10	1.714e+09	2.015	0.0861
	Residuals	30	2.552e+10	8.507e+08		
	Age (HWRC)	2	132581682	66290841	1.375	0.322
	Residuals	6	289290548	48215091		
Klebsiella	Habitat	2	29537442	14768721	1.18	0.319
	Residuals	36	450556373	12515455		
	SubHabitat	5	76874617	15374923	1.222	0.321
	Residuals	32	402569452	12580295		

	Site	7	275257457	39322494	5.777	0.000266***
	Residuals	30	204186612	6806220		
	Age (HWRC)	2	10590	5295	2.513	0.161
	Residuals	6	12644	2107		
Yersinia	Habitat	2	2436	1218	1.145	0.33
	Residuals	36	38314	1064		
	SubHabitat	5	4354	870.9	0.767	0.581
	Residuals	32	36341	1135.7		
	Site	7	4114	587.7	0.482	0.84
	Residuals	30	36581	1219.4		
	Age (HWRC)	2	728.4	364.2	2.332	0.178
	Residuals	6	937.2	156.2		
Mycoplasma	Habitat	2	3347	1673.3	3.112	0.0567
	Residuals	36	19358	537.7		
	SubHabitat	5	4321	864.1	2.557	0.0469*
	Residuals	32	10816	338.0		
	Site	7	9136	1305.2	2.949	0.0178*
	Residuals	30	13276	442.5		
	Age (HWRC)	2	0.1389	0.06944	0.556	0.601
	Residuals	6	0.7500	0.12500		

Table S6. Tukey's Honest Significant Difference (HSD) test results - Genus

Streptococcus ~ Habitat

	diff	lwr	upr	*p adj*
SubUrban-Captive	19578.4957	-3845.766	4.30E+04	0.1165402
Urban-Captive	-323.2418	-22591.613	2.19E+04	0.9993062
Urban-SubUrban	-19901.738	-39804.437	9.62E-01	0.0500132

Mycoplasma ~ Habitat

	diff	lwr	upr	*p adj*
SubUrban-Captive	1.504274	-23.073905	2.61E+01	0.9877418
Urban-Captive	19.535948	-3.8294	4.29E+01	0.1163795
Urban-SubUrban	18.031674	-2.851464	3.89E+01	0.1018497

Ligilactobacillus ~ Habitat

	diff	lwr	upr	*p adj*
SubUrban-Captive	-15403.64	-70637.253	3.98E+04	0.7755946
Urban-Captive	33692.02	-18816.043	8.62E+04	0.2721786
Urban-SubUrban	49095.66	2165.772	9.60E+04	0.0386899*

Acinetobacter ~ Habitat

	diff	lwr	upr	*p adj*
SubUrban-Captive	-3443.2393	-7332.749	4.46E+02	0.0913676
Urban-Captive	-3396.5425	-7094.121	3.01E+02	0.0771029
Urban-SubUrban	46.69683	-3258.071	3.35E+03	0.9993426

Acinetobacter ~ SubHabitat

	diff	lwr	upr	*p adj*
Coast-City	-159.66667	-5195.418	4876.0847	0.9999987
DayOne-City	-148.25	-6567.5986	6271.0986	0.9999997
Lake-City	-90.857143	-5474.3018	5292.5875	0.9999999
LongTerm-City	6097.8	139.41862	12056.1814	0.042423*
PowerStation-City	-155.75	-6575.0986	6263.5986	0.9999997
DayOne-Coast	11.416667	-6407.932	6430.7653	1
Lake-Coast	68.809524	-5314.6351	5452.2542	1
LongTerm-Coast	6257.46667	299.08529	12215.848	0.0350284*
PowerStation-Coast	3.916667	-6415.432	6423.2653	1
Lake-DayOne	57.392857	-6638.1819	6752.9676	1
LongTerm-DayOne	6246.05	-919.94983	13412.0498	0.1166205
PowerStation-DayOne	-7.5	-7561.1271	7546.1271	1
LongTerm-Lake	6188.65714	-66.34031	12443.6546	0.0538163
PowerStation-Lake	-64.892857	-6760.4676	6630.6819	1
PowerStation-LongTerm	-6253.55	-13419.55	912.4498	0.1158659

Mycoplasma ~ SubHabitat

	diff	lwr	upr	*p adj*
Coast-City	22.88889	-3.36341	49.141187	0.1164338
DayOne-City	-2.75	-36.21525	30.715245	0.9998561
Lake-City	-3	-31.06489	25.064887	0.9994783
LongTerm-City	-3	-34.06214	28.062138	0.999682
PowerStation-City	-3	-36.46525	30.465245	0.9997793
DayOne-Coast	-25.63889	-59.10413	7.826357	0.2154862
Lake-Coast	-25.88889	-53.95378	2.175999	0.0843814
LongTerm-Coast	-25.88889	-56.95103	5.173249	0.1470102
PowerStation-Coast	-25.88889	-59.35413	7.576357	0.2068923
Lake-DayOne	-0.25	-35.15526	34.655263	1
LongTerm-DayOne	-0.25	-37.60768	37.107676	1
PowerStation-DayOne	-0.25	-39.62845	39.128448	1
LongTerm-Lake	0	-32.60845	32.608453	1
PowerStation-Lake	6.66134E-16	-34.90526	34.905263	1
PowerStation-LongTerm	6.66134E-16	-37.35768	37.357676	1

Bacillus ~ Site

	diff	lwr	upr	*p adj*
Gloucester-Bristol	309.1666667	-46.27369	664.60703	0.1250217
Hereford Wildlife Rescue Centre-Bristol	0.6111111	-303.76983	304.99205	1
Hinkley Point-Bristol	-4.5	-341.70033	332.70033	1
Liverpool-Bristol	21	-286.82038	328.82038	0.9999983
Portishead-Bristol	68.5	-243.68685	380.68685	0.9958929
West Kirby-Bristol	-7.5	-396.86541	381.86541	1
Weston Super-Mare-Bristol	21.8333333	-333.60703	377.27369	0.9999992
Hereford Wildlife Rescue Centre-Gloucester	-308.5555556	-568.13249	-48.97862	0.0112731*
Hinkley Point-Gloucester	-313.6666667	-611.04941	-16.28392	0.0329943*
Liverpool-Gloucester	-288.1666667	-551.76829	-24.56504	0.024429*
Portishead-Gloucester	-240.6666667	-509.35432	28.02099	0.1051431
West Kirby-Gloucester	-316.6666667	-672.10703	38.77369	0.1086114
Weston Super-Mare-Gloucester	-287.3333333	-605.24886	30.58219	0.0995054
Hinkley Point-Hereford Wildlife Rescue Centre	-5.1111111	-239.0906	228.86838	1
Liverpool-Hereford Wildlife Rescue Centre	20.3888889	-168.80869	209.58647	0.9999605
Portishead-Hereford Wildlife Rescue Centre	67.8888889	-128.33283	264.11061	0.9458083
West Kirby-Hereford Wildlife Rescue Centre	-8.1111111	-312.49205	296.26983	1
Weston Super-Mare-Hereford Wildlife Rescue Centre	21.2222222	-238.35472	280.79916	0.999994
Liverpool-Hinkley Point	25.5	-212.93664	263.93664	0.9999625
Portishead-Hinkley Point	73	-171.04768	317.04768	0.9748793
West Kirby-Hinkley Point	-3	-340.20033	334.20033	1
Weston Super-Mare-Hinkley Point	26.3333333	-271.04941	323.71608	0.9999896
Portishead-Liverpool	47.5	-154.01574	249.01574	0.9936503
West Kirby-Liverpool	-28.5	-336.32038	279.32038	0.9999859
Weston Super-Mare-Liverpool	0.8333333	-262.76829	264.43496	1
West Kirby-Portishead	-76	-388.18685	236.18685	0.9922962
Weston Super-Mare-Portishead	-46.6666667	-315.35432	222.02099	0.9990596
Weston Super-Mare-West Kirby	29.3333333	-326.10703	384.77369	0.9999936

Mycoplasma ~ Site

	diff	lwr	upr	*p adj*
Gloucester-Bristol	-0.5	-62.981997	61.981997	1
Hereford Wildlife Rescue Centre-Bristol	-0.3888889	-53.895275	53.117497	1
Hinkley Point-Bristol	-0.5	-59.775627	58.775627	1
Liverpool-Bristol	37.875	-16.235997	91.985997	0.3380117
Portishead-Bristol	-0.5	-55.378567	54.378567	1
West Kirby-Bristol	10	-58.445598	78.445598	0.9996961
Weston Super-Mare-Bristol	-0.5	-62.981997	61.981997	1
Hereford Wildlife Rescue Centre-Gloucester	0.1111111	-45.519288	45.74151	1
Hinkley Point-Gloucester	-4.44089E-16	-52.276189	52.276189	1
Liverpool-Gloucester	38.375	-7.962889	84.712889	0.1633913
Portishead-Gloucester	2.53765E-16	-47.23195	47.23195	1
West Kirby-Gloucester	10.5	-51.981997	72.981997	0.9992402
Weston Super-Mare-Gloucester	5.32907E-15	-55.885597	55.885597	1
Hinkley Point-Hereford Wildlife Rescue Centre	-0.1111111	-41.241797	41.019575	1
Liverpool-Hereford Wildlife Rescue Centre	38.26389	5.005306	71.522472	0.0154224*
Portishead-Hereford Wildlife Rescue Centre	-0.1111111	-34.604451	34.382228	1
West Kirby-Hereford Wildlife Rescue Centre	10.38889	-43.117497	63.895275	0.9980838
Weston Super-Mare-Hereford Wildlife Rescue Centre	-0.1111111	-45.74151	45.519288	1
Liverpool-Hinkley Point	38.375	-3.539198	80.289198	0.0917735
Portishead-Hinkley Point	6.97855E-16	-42.900549	42.900549	1
West Kirby-Hinkley Point	10.5	-48.775627	69.775627	0.9989324
Weston Super-Mare-Hinkley Point	5.77316E-15	-52.276189	52.276189	1
Portishead-Liverpool	-38.375	-73.798963	-2.951037	0.0264091*
West Kirby-Liverpool	-27.875	-81.985997	26.235997	0.7016972
Weston Super-Mare-Liverpool	-38.375	-84.712889	7.962889	0.1633913
West Kirby-Portishead	10.5	-44.378567	65.378567	0.9982538
Weston Super-Mare-Portishead	5.07531E-15	-47.23195	47.23195	1
Weston Super-Mare-West Kirby	-10.5	-72.981997	51.981997	0.9992402

Ligilactobacillus ~ Site

	diff	lwr	upr	*p adj*
Gloucester-Bristol	-132619.1667	-265145.24	-93.09123	0.0497391*
Hereford Wildlife Rescue Centre-Bristol	-112674.1667	-226162.72	814.38412	0.0527364
Hinkley Point-Bristol	-132152	-257877.27	-6426.7257	0.0339349*
Liverpool-Bristol	-78145.75	-192916.7	36625.198	0.3713237
Portishead-Bristol	-124247.9286	-240646.91	-7848.9426	0.0299034*
West Kirby-Bristol	-133334	-278509.04	11841.042	0.0899628
Weston Super-Mare-Bristol	-117218.5	-249744.58	15307.5754	0.1134018
Hereford Wildlife Rescue Centre-Gloucester	19945	-76838.36	116728.361	0.9972147
Hinkley Point-Gloucester	467.1667	-110412.1	111346.436	1
Liverpool-Gloucester	54473.4167	-43810.55	152757.385	0.6227379
Portishead-Gloucester	8371.2381	-91809.06	108551.535	0.999993
West Kirby-Gloucester	-714.8333	-133240.91	131811.242	1
Weston Super-Mare-Gloucester	15400.6667	-103134.26	133935.592	0.9998607
Hinkley Point-Hereford Wildlife Rescue Centre	-19477.8333	-106717.18	67761.5096	0.995431
Liverpool-Hereford Wildlife Rescue Centre	34528.4167	-36013.97	105070.807	0.7508387
Portishead-Hereford Wildlife Rescue Centre	-11573.7619	-84735.11	61587.5824	0.9994874
West Kirby-Hereford Wildlife Rescue Centre	-20659.8333	-134148.38	92828.7175	0.9987281
Weston Super-Mare-Hereford Wildlife Rescue Centre	-4544.3333	-101327.69	92239.028	0.9999999
Liverpool-Hinkley Point	54006.25	-34894.94	142907.444	0.513051
Portishead-Hinkley Point	7904.0714	-83089.2	98897.3445	0.9999909
West Kirby-Hinkley Point	-1182	-126907.27	124543.274	1
Weston Super-Mare-Hinkley Point	14933.5	-95945.77	125812.77	0.9998229
Portishead-Liverpool	-46102.1786	-121237.4	29033.0438	0.5006565
West Kirby-Liverpool	-55188.25	-169959.2	59582.698	0.7666339
Weston Super-Mare-Liverpool	-39072.75	-137356.72	59211.218	0.8941885
West Kirby-Portishead	-9086.0714	-125485.06	107312.915	0.9999956
Weston Super-Mare-Portishead	7029.4286	-93150.87	107209.725	0.9999979
Weston Super-Mare-West Kirby	16115.5	-116410.58	148641.575	0.9999106

Klebsiella ~ Site

	diff	lwr	upr	*p adj*
Gloucester-Bristol	9888.167	2139.29	17637.044	0.0054085**
Hereford Wildlife Rescue Centre-Bristol	-101.1667	-6736.909	6534.575	1
Hinkley Point-Bristol	-17.25	-7368.48	7333.98	1
Liverpool-Bristol	-116.375	-6827.099	6594.349	1
Portishead-Bristol	-108.0714	-6913.988	6697.845	1
West Kirby-Bristol	-120	-8608.47	8368.47	1
Weston Super-Mare-Bristol	-101.8333	-7850.71	7647.044	1
Hereford Wildlife Rescue Centre-Gloucester	-9989.333	-15648.313	-4330.354	0.0000709***
Hinkley Point-Gloucester	-9905.417	-16388.592	-3422.241	0.0005962***
Liverpool-Gloucester	-10004.54	-15751.263	-4257.821	0.0000882***
Portishead-Gloucester	-9996.238	-15853.839	-4138.638	0.0001201***
West Kirby-Gloucester	-10008.17	-17757.044	-2259.29	0.0047382**
Weston Super-Mare-Gloucester	-9990	-16920.806	-3059.194	0.0012849**
Hinkley Point-Hereford Wildlife Rescue Centre	83.91667	-5017.019	5184.852	1
Liverpool-Hereford Wildlife Rescue Centre	-15.20833	-4139.863	4109.446	1
Portishead-Hereford Wildlife Rescue Centre	-6.904762	-4284.691	4270.882	1
West Kirby-Hereford Wildlife Rescue Centre	-18.83333	-6654.575	6616.909	1
Weston Super-Mare-Hereford Wildlife Rescue Centre	-0.6666667	-5659.646	5658.313	1
Liverpool-Hinkley Point	-99.125	-5297.23	5098.98	1
Portishead-Hinkley Point	-90.82143	-5411.251	5229.608	1
West Kirby-Hinkley Point	-102.75	-7453.98	7248.48	1
Weston Super-Mare-Hinkley Point	-84.58333	-6567.759	6398.592	1
Portishead-Liverpool	8.303571	-4384.897	4401.504	1
West Kirby-Liverpool	-3.625	-6714.349	6707.099	1
Weston Super-Mare-Liverpool	14.54167	-5732.179	5761.263	1
West Kirby-Portishead	-11.92857	-6817.845	6793.988	1
Weston Super-Mare-Portishead	6.238095	-5851.362	5863.839	1
Weston Super-Mare-West Kirby	18.16667	-7730.71	7767.044	1

Table S7. ANOVA test results – Alpha Diversity

ANOVA results for alpha diversity indexes. Significant p-values marked with "*" for p<0.05, "**" for p<0.01 and "***" for p<0.001.

Diversity		D.f.	Sum Sq	Mean sq	F value	*P*
Species Richness	Habitat	2	181380	90690	3.242	0.0507
	Residuals	36	1006980	27972		
	SubHabitat	5	220162	44032	1.459	0.231
	Residuals	32	965705	30178		
	Site	7	267607	38230	1.249	0.308
	Residuals	30	918112	30604		
	Age (HWRC)	2	54921	27460	0.33	0.731
	Residuals	6	499273	83212		
Shannon	Habitat	2	3.278	1.6392	3.878	0.0298*
	Residuals	36	15.217	0.4227		
	SubHabitat	5	3.872	0.7744	1.83	0.135
	Residuals	32	13.540	0.4231		
	Site	7	4.873	0.6962	1.628	0.166
	Residuals	30	12.832	0.4277		
	Age (HWRC)	2	1.012	0.5059	0.705	0.531
	Residuals	6	4.304	0.7173		
Pielou	Habitat	2	0.0521	0.02607	1.971	0.154
	Residuals	36	0.4762	0.01323		
	SubHabitat	5	0.0701	0.01402	1.091	0.384
	Residuals	32	0.4112	0.01285		
	Site	7	0.1277	0.01825	1.497	0.206
	Residuals	30	0.3658	0.01219		
	Age (HWRC)	2	0.02142	0.01071	0.875	0.464
	Residuals	6	0.07347	0.01224		
OTU Count per sample	Habitat	2	4.585e+09	2.292e+09	0.587	0.561
	Residuals	36	1.406e+11	3.906e+09		
	SubHabitat	5	3.506e+10	7.012e+09	2.107	0.0902
	Residuals	32	1.065e+11	3.329e+09		
	Site	7	3.477e+10	4.968e+09	1.35	0.262
	Residuals	30	1.104e+11	3.681e+09		
	Age (HWRC)	2	1.640e+09	8.198e+08	0.207	0.819
	Residuals	6	2.377e+10	3.962e+09		
Simpson	Habitat	2	0.2157	0.10783	2.55	0.0921
	Residuals	36	1.5223	0.04229		
	SubHabitat	5	0.2242	0.04483	1.102	0.378
	Residuals	32	1.3015	0.04067		
	Site	7	0.4408	0.06297	1.65	0.16
	Residuals	30	1.1451	0.03817		
	Age (HWRC)	2	0.05071	0.02535	0.991	0.425
	Residuals	6	0.15352	0.02559		
Inverse Simpson	Habitat	2	71.2	35.61	1.588	0.218
	Residuals	36	807.3	22.42		

	SubHabitat	5	75.7	15.14	0.617	0.688
	Residuals	32	785.8	24.56		
	Site	7	390.1	55.73	3.449	0.00794**
	Residuals	30	484.8	16.16		
	Age (HWRC)	2	0.4166	0.2083	1.292	0.341
	Residuals	6	0.9670	0.1612		
Effective Number of Species	Habitat	2	215.5	107.74	4.073	0.0254*
	Residuals	36	952.3	26.45		
	SubHabitat	5	305.6	61.12	2.314	0.0667
	Residuals	32	845.2	26.41		
	Site	7	241.5	34.50	1.136	0.368
	Residuals	30	911.3	30.38		
	Age (HWRC)	2	112.8	56.42	0.613	0.573
	Residuals	6	552.5	92.09		
Berger Parker Index	Habitat	2	0.1592	0.07960	2.22	0.123
	Residuals	36	1.2908	0.03586		
	SubHabitat	5	0.2124	0.04249	1.268	0.302
	Residuals	32	1.0723	0.03351		
	Site	7	0.3289	0.04698	1.428	0.231
	Residuals	30	0.9872	0.03291		
	Age (HWRC)	2	0.06777	0.03389	1.431	0.31
	Residuals	6	0.14212	0.02369		

Table S8. Tukey's Honest Significant Difference (HSD) test results – Alpha Diversity

Shannon ~ Habitat

	diff	lwr	upr	*p adj*
SubUrban-Captive	-0.69589744	-1.3850055	-0.0067894	0.0473665*
Urban-Captive	-0.68196078	-1.3370642	-0.0268574	0.0398284*
Urban-SubUrban	0.01393665	-0.5715721	0.59944542	0.9981356

Effective number of Species ~ Habitat

	diff	lwr	upr	*p adj*
SubUrban-Captive	-6.0096581	-11.460918	-0.5583979	0.028017*
Urban-Captive	-5.1135948	-10.295858	0.06866842	0.0537439
Urban-SubUrban	0.8960633	-3.735664	5.52779082	0.8844368

Inverse Simpson ~ Site

	diff	lwr	upr	*p adj*
Gloucester-Bristol	-14.48666667	-26.426403	-2.5469304	0.0091862**
Hereford Wildlife Rescue Centre-Bristol	-14.43333333	-24.657913	-4.2087541	0.0016701**
Hinkley Point-Bristol	-14.1575	-25.484528	-2.8304717	0.0067554**
Liverpool-Bristol	-12.40625	-22.746365	-2.0661351	0.0102794*

Portishead-Bristol	-14.34	-24.826791	-3.8532095	0.0024592**
West Kirby-Bristol	-13.965	-27.044326	-0.8856743	0.0298373*
Weston Super-Mare-Bristol	-14.35666667	-26.296403	-2.4169304	0.0100588*
Hereford Wildlife Rescue Centre-Gloucester	0.05333333	-8.666217	8.7728838	1
Hinkley Point-Gloucester	0.32916667	-9.660333	10.3186667	1
Liverpool-Gloucester	2.08041667	-6.774329	10.935162	0.9937733
Portishead-Gloucester	0.14666667	-8.878926	9.1722589	1
West Kirby-Gloucester	0.52166667	-11.41807	12.4614029	0.9999999
Weston Super-Mare-Gloucester	0.13	-10.549225	10.8092247	1
Hinkley Point-Hereford Wildlife Rescue Centre	0.27583333	-7.583863	8.1355299	1
Liverpool-Hereford Wildlife Rescue Centre	2.02708333	-4.328327	8.3824933	0.9644636
Portishead-Hereford Wildlife Rescue Centre	0.09333333	-6.498027	6.6846939	1
West Kirby-Hereford Wildlife Rescue Centre	0.46833333	-9.756246	10.6929126	0.9999999
Weston Super-Mare-Hereford Wildlife Rescue Centre	0.07666667	-8.642884	8.7962172	1
Liverpool-Hinkley Point	1.75125	-6.258169	9.7606686	0.9959808
Portishead-Hinkley Point	-0.1825	-8.380401	8.0154013	1
West Kirby-Hinkley Point	0.1925	-11.134528	11.5195283	1
Weston Super-Mare-Hinkley Point	-0.19916667	-10.188667	9.7903334	1
Portishead-Liverpool	-1.93375	-8.702944	4.8354442	0.9805425
West Kirby-Liverpool	-1.55875	-11.898865	8.7813649	0.9996264
Weston Super-Mare-Liverpool	-1.95041667	-10.805162	6.9043287	0.9957945
West Kirby-Portishead	0.375	-10.111791	10.8617905	1
Weston Super-Mare-Portishead	-0.01666667	-9.042259	9.0089256	1
Weston Super-Mare-West Kirby	-0.39166667	-12.331403	11.5480696	1

Figure S1. Species Richness Curves

Library 1:

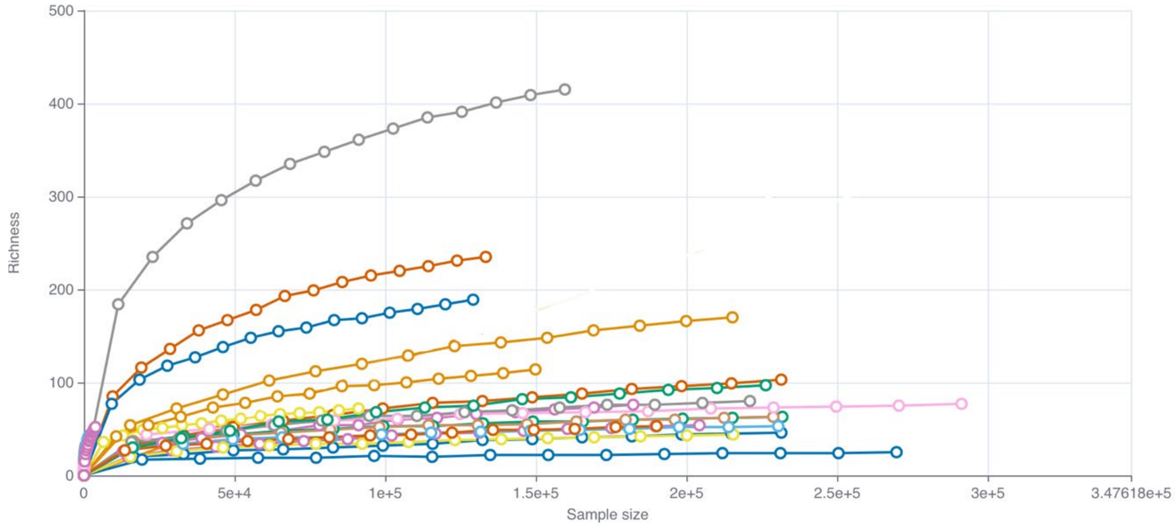

Library 2:

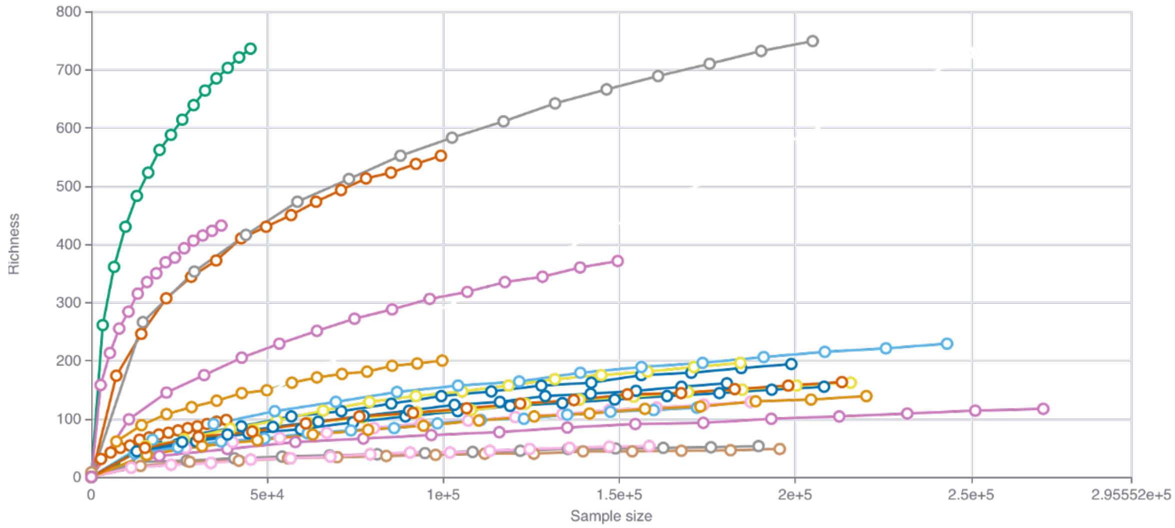

Supplement: Supplementary file 1 [file animals-16-00300-s001.zip › animals-4044506-supplementary.pdf]
